# Variations in hydrodynamic characteristics of netting panels with various twine materials, knot types, and weave patterns at small attack angles

**DOI:** 10.1038/s41598-018-35907-1

**Published:** 2019-02-13

**Authors:** Hao Tang, Fuxiang Hu, Liuxiong Xu, Shuchuang Dong, Cheng Zhou, Xuefang Wang

**Affiliations:** 1 College of Marine Sciences, Shanghai Ocean University, Shanghai, 201306 P. R. China; 2 National Engineering Research Center for Oceanic Fisheries, Shanghai, 201306 P. R. China; 3 Key Laboratory of Oceanic Fisheries Exploration, Ministry of Agriculture and Rural Affairs, Shanghai, 201306 China; 4 The Key Laboratory of Sustainable Exploitation of Oceanic Fisheries Resources, Shanghai Ocean University, Ministry of Education, Shanghai, 201306 China; 5 Scientific Observing and Experimental Station of Oceanic Fishery Resources, Ministry of Agriculture and Rural Affairs, Shanghai, 201306 China; 6 Faculty of Marine Science, Tokyo University of Marine Science and Technology, Minato, Tokyo 108-8477 Japan

## Abstract

It is essential to conduct hydrodynamic experiments for fishing gear at small attack angles along the flow direction to better understand the hydrodynamic characteristics of netting and application of gear. The hydrodynamic characteristics of netting panels made of different materials at small attack angles were investigated by a self-designed setup; this is essential for the effective use of netting on different types of gears. As confirmed by experiments, the measured drag of designed frame without netting accounted for less than 20% of the total setup drag including experimental netting and remained in a steady state under various current speeds and small attack angles, indicating that the self-designed frame setup is suitable for such trials. The drag coefficient was determined by varying the attack angle, solidity ratio, Reynolds number, knot types, weave pattern, and twine materials at small attack angles. The results indicate that the drag coefficient increased as the attack angle increased, but decreased as the solidity ratio and Reynolds number increased. The drag generated by knot accounted for 21% of the total drag of nylon (PA) netting. For braided knotless netting, the drag coefficient of PA netting was about 8.4% lower than that of polythene netting (PE) and 7% lower than that of polyester netting (PES). Compared with twined netting, the braided netting exhibited a higher resistance to flow, corresponding to higher values of drag coefficient.

## Introduction

A good understanding of hydrodynamic characteristics of fishing gear is essential for the development and operation of efficient gears with a low impact on environment and nontarget species. As the main component of a fishing gear, the hydrodynamic characteristics of a netting determine whether its modification and development are successful or not. Most previous studies evaluated fabric nets as a fishing gear material because it is difficult to predict how the nets change with ever-changing sea conditions. For a netting panel, drag depends on the netting area, flow velocity, solidity, Reynolds number, and attack angle; drag is affected by knot type, weave pattern, twine bending stiffness^1^, and netting-surface roughness^2,3^.

Extensive studies have been conducted on fishing gear consisting of fabric nets focusing on the hydrodynamic characteristics of fabric itself^4–8^ Aarsnes *et al*.^9^ derived a formula for drag coefficient as a function of solidity ratio (the ratio of twine diameter to the bar length) and angle of incidence (angle between the current direction and normal to a net plane) based on tow tank data; the effect of various attack angles was not considered. Zhan *et al*.^5^ reported that the drag coefficient could be determined from solidity and current speed. Balash *et al*.^6^ performed experimental studies and proposed a corrected formula for drag coefficient. Hosseini *et al*.^7^ obtained the drag and lift coefficients of nylon (PA) knotted netting from flume tank experiments to simulate the sinking behavior of purse seine; the hydrodynamic coefficients depend on Reynolds number and attack angle. Kumazawa *et al*.^8^ evaluated the hydrodynamic characteristics of knotless netting panels composed of three materials (Dyneema, polyamide, and polyvinyl) through flume tank experiments. Zhou *et al*.^10^ and Tang *et al*.^3^ investigated the drag and lift coefficients of netting at an attack angle ranging from 0° to 90° with 10° interval; the drag coefficient rapidly changed at small attack angles, making the prediction difficult. Tang *et al*.^11^ investigated the differences in hydrodynamic coefficients between knotless and knotted PA netting panels by setting normal to the flow 1.23–1.35 times higher than that of knotless netting; a remarkable difference was found in the lift coefficient between different netting types at small attack angles.

Generally, when estimating gear performance, the abovementioned formula had problems with variable results, lack of results for small attack angles, and nondeterministic solidity ratio. Numerous studies have shown that the force acting on a net is directly proportional to the drag coefficient. Thus, small differences in drag coefficient can lead to large differences in force, and while evaluating the forces acting on a net, the effect of attack angle cannot be ignored, especially small attack angles.

Because the underwater shape is affected by current, fishing gear structures, and fishing operations, the movement of fishing gear is shown in an approximate angle state along the current direction. For a trawl, the angle of inclination between netting and flow is generally less than 15° during relatively stable towing^11^. In purse seine fishing operations, the small-attack-angle movement of netting along the current direction is a common phenomenon during shooting and sinking. The accurate measurement and prediction of gear hydrodynamic performance during the development using water-tank experiment technique are most important and challenging subjects in the field of gear hydrodynamics. However, still some problems should be solved to improve the hydrodynamic performance of a netting and promote its practical use, such as the relationship between small attack angles and its resistance. However, the progress in studies of hydrodynamic characteristics of a netting at small attack angles is slow because of the limitations of experimental setup including the frame, installation methods, and precision instrument.

The aim of this study is to validate a self-designed setup, evaluate the hydrodynamic characteristics of netting panels made of three twine materials (polythene (PE) netting, polyester (PES) netting, PA netting), and quantify the effect of knot, weave pattern (twined and braided), and twine material on the drag coefficient at small attack angles by flume tank experiments. These results can help to determine the drag coefficient of netting, providing more accurate estimation of dynamic characteristics of fishing gear and highlighting the differences in hydrodynamic characteristics of different nettings materials at small attack angles.

## Results

### Effectiveness of experimental frame

**Figure 1:**
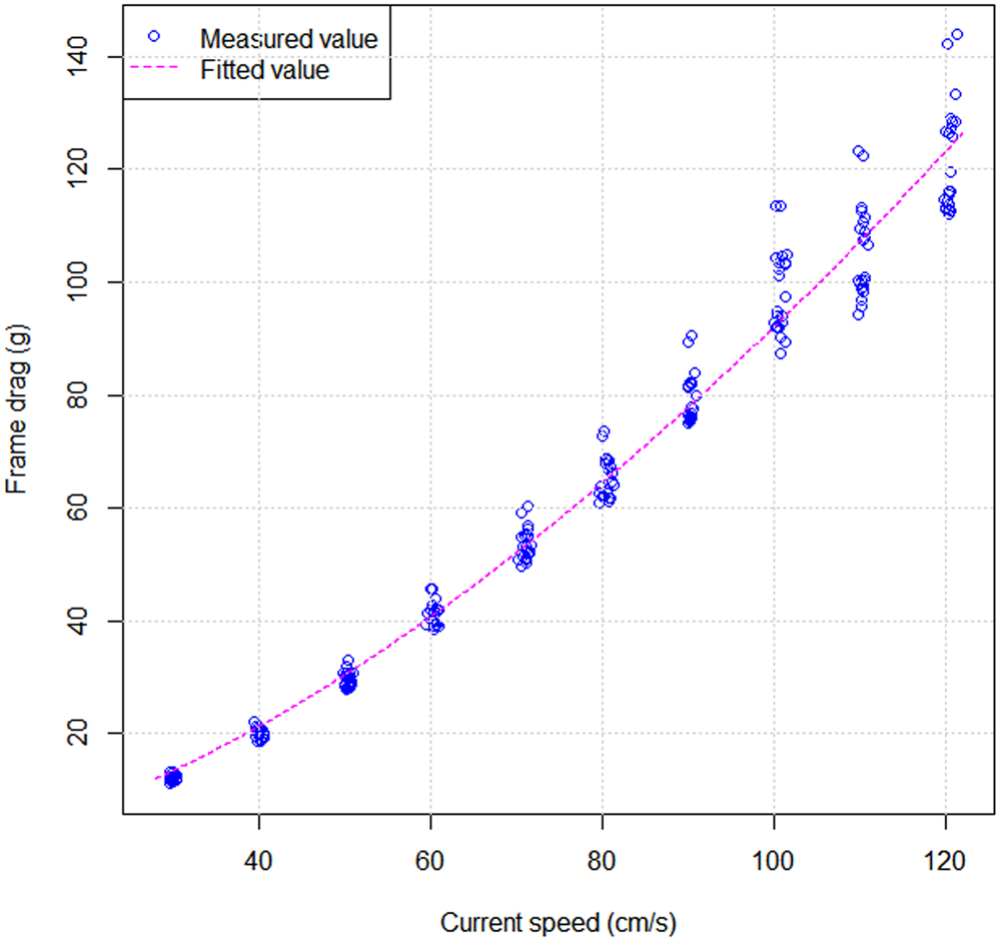
Realationship between experimental frame drag and current speed.

To verify the effectiveness of experimental frame, the frame drags were measured at different current speeds and attack angles. The results indicate that the frame drag increased as the current speed increased; their relationships were exponential regression (Fig. 1). Figure 1 shows that the frame drags are only 11 g and 120 g at a current speed of 30 cm/s and 120 cm/s, respectively. A small fluctuation occurred at a higher current speed (100–120 cm/s) on frame drag.

Using nonlinear regression with the data argument predicted from the formula of frame drags, the following expression can be proposed:

1 $${D}_{frame}=0.059\times {{V}_{current}}^{1.595}$$

where, *D*_*frame*_ is the frame drag; *V*_*current*_ is the current speed.

**Figure 2:**
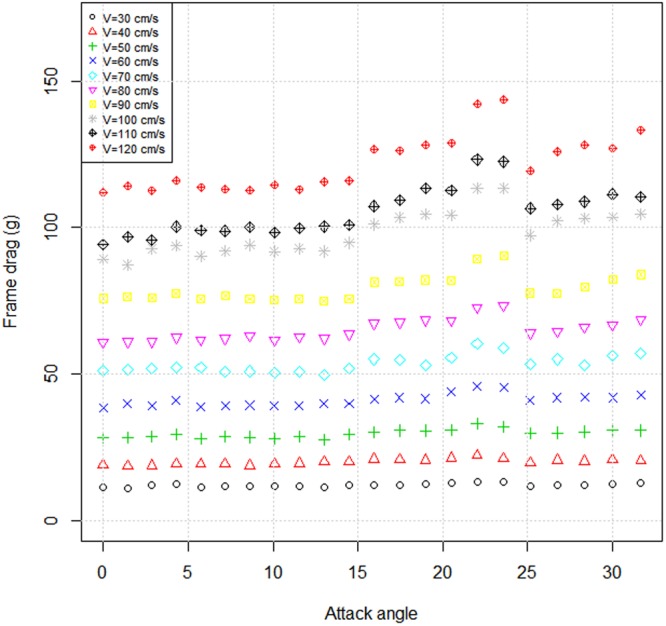
Experimental frame drags with respect to attack angles at various current speeds.

The experimental results of frame drag at different attack angles (0° < *θ* < 31°) subjected to current are as follows: As the attack angle increased, the frame drag had slight significant change at the same current speed. However, the drag clearly fluctuated at attack angles ranging from 20° to 30°, and the amplitude of fluctuation depends on the current speed (Fig. 2). The frame lift was also measured at different attack angles and current speeds to determine the behavior of two streamlined planes of frame set parallel to the incoming flow. The frame lift is typically distributed at about 0, except in the case of higher current speeds (>100 cm/s) and large attack angels (>20°). Clearly, the designed experimental frame can be used to evaluate the hydrodynamic characteristics of netting panels at attack angles ranging from 0° to 20°.

### Effect of solidity ratio

**Figure 3:**
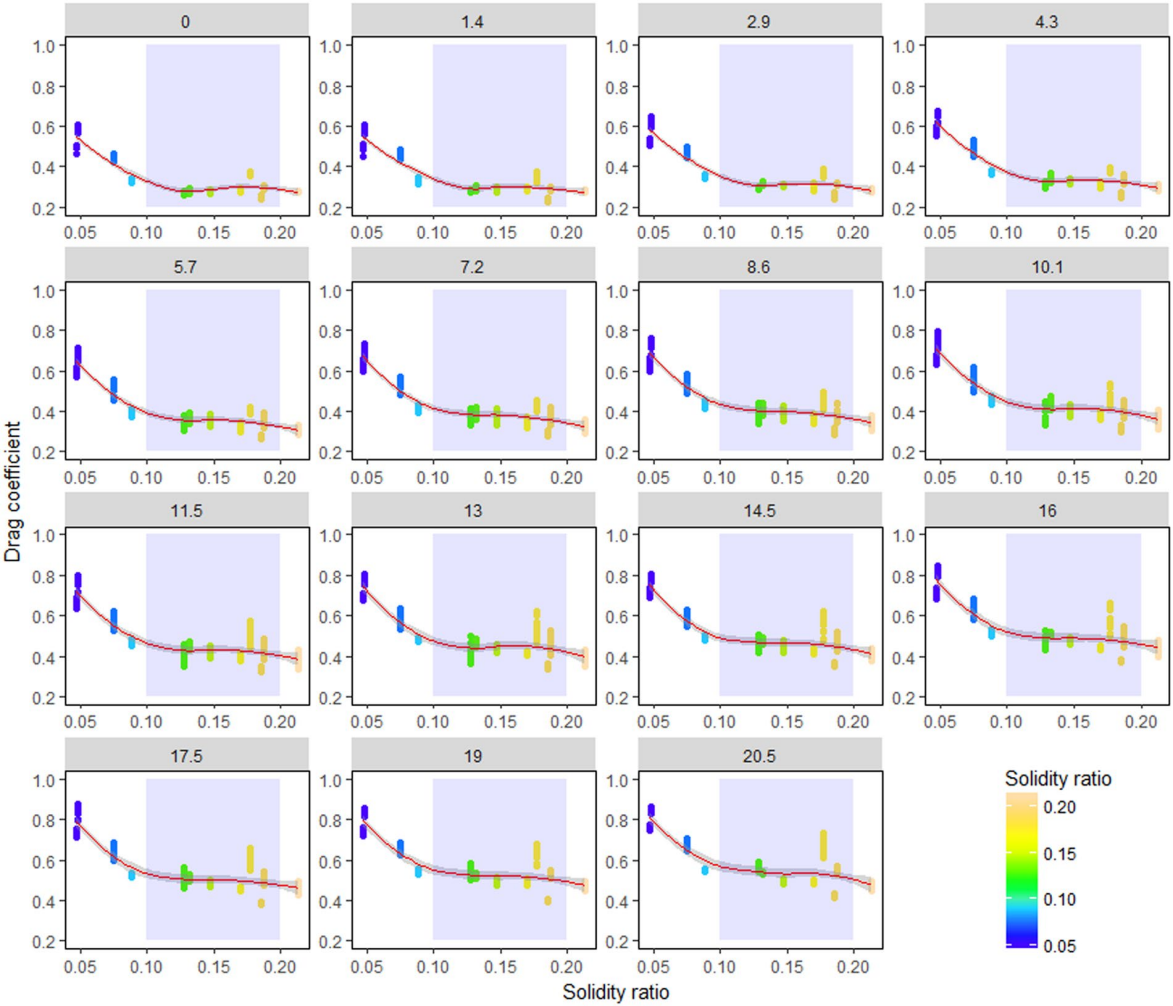
Effect of solidity ratio on drag coefficient and their interactions, including 95% confidence intervals. *Red lines* represent smooth curves; *shadow sections* show the standard-error confidence intervals.

Solidity ratio is characterized by mesh size, twine diameter, and opening angle (hanging ratio) that determine the geometric characteristics of netting. The drag coefficient of all netting panels with a lower solidity ratio was higher than that with a higher solidity ratio (Fig. 3), whereas the drag coefficient became stable when the solidity ratio ranged from 0.1 to 0.2, as shown in the blue sections of Fig. 3. The results show that the large fluctuation of smooth curves occurs at a larger attack angle, indicating that the effect of solidity ratio on drag coefficient gradually increases with the increase in attack angle.

### Effect of Reynolds number

**Figure 4:**
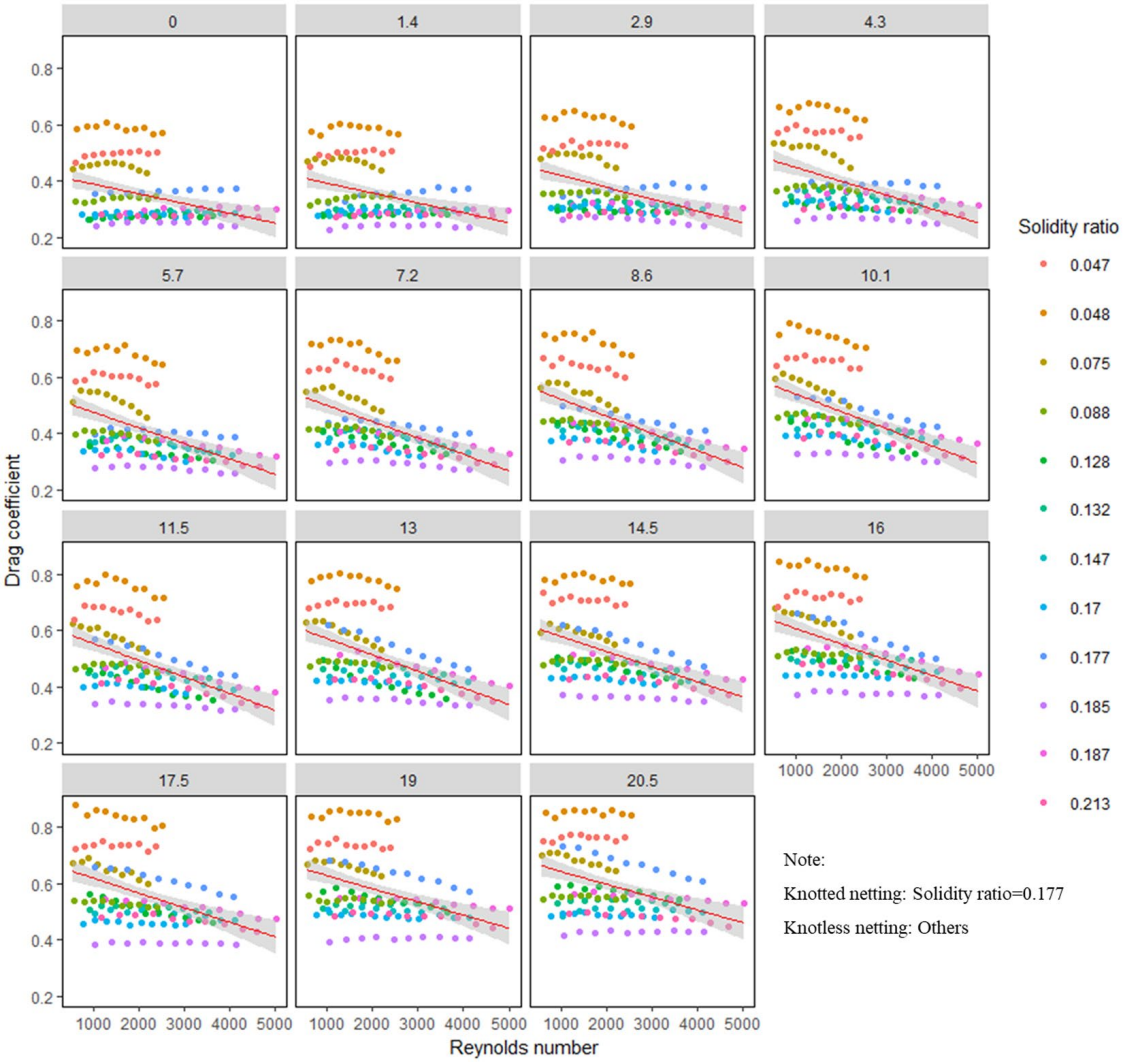
Effect of Reynolds number on drag coefficient and their interactions, including 95% confidence intervals. *Red lines* represent smooth curves; *shadow sections* show the standard-error confidence intervals.

An increase in Reynolds number by changing the flow changes the characteristics of flow regime in flume. The experimental results indicate that the drag coefficient rapidly changed when the Reynolds number was less than 2000. The drag coefficient decreased as the Reynolds number increased until a certain value, and then the decrease became limited (Fig. 4). For the same type of netting panel (twine material, knot type, and weave pattern), the effect of Reynolds number on drag coefficient increased with increasing attack angle.

### Effect of twine materials and weave patterns

**Figure 5:**
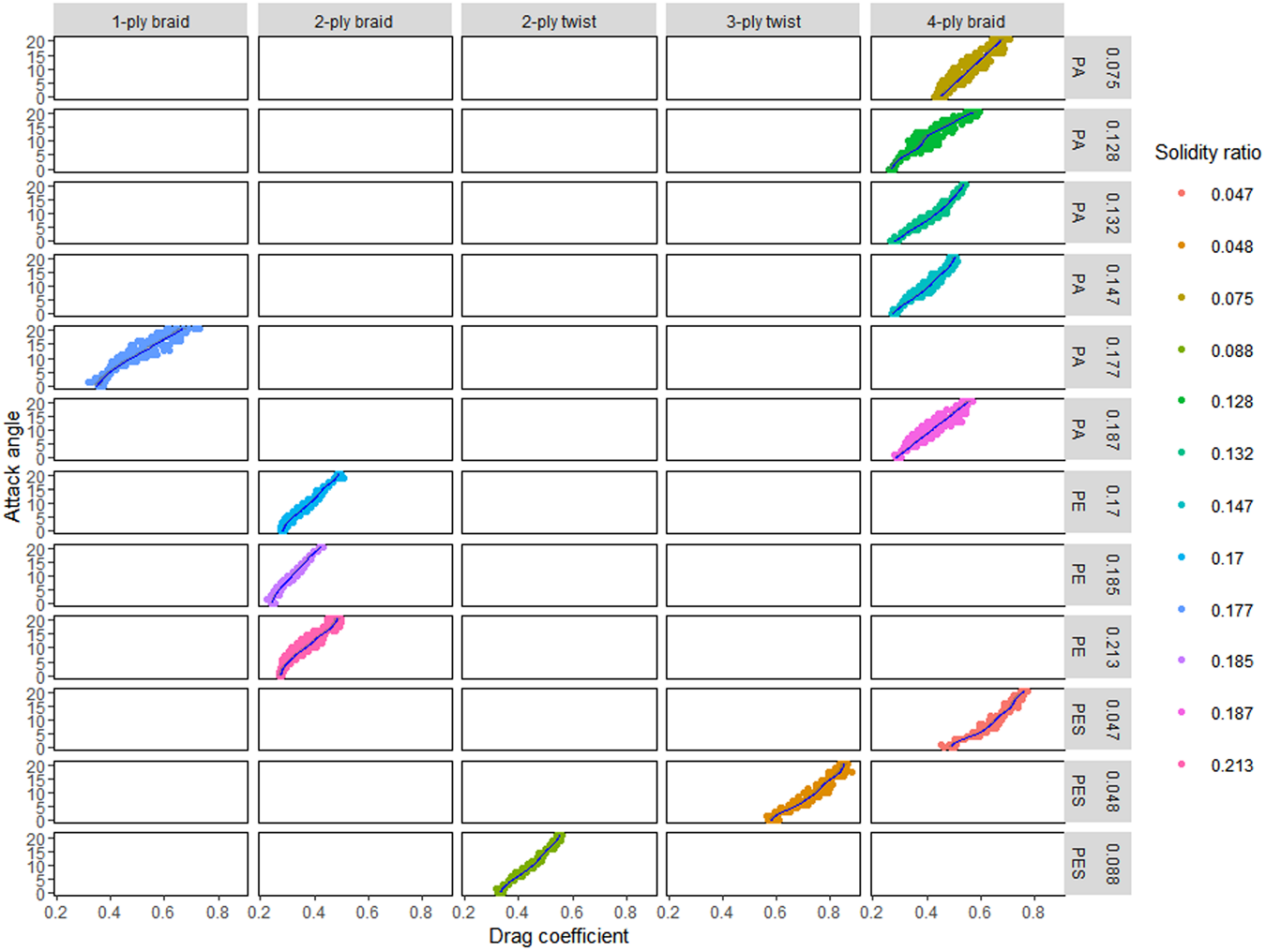
Comparison of drag coefficients of netting panels with different solidity ratios and attack angles.

Figure 5 shows the drag coefficient of each netting sample as a function of small attack angles. The blue lines show the fitted curve between attack angles and drag coefficients. The nettings made of different materials have various hydrodynamic characteristics depending on the weave pattern and attack angle. For PA netting, the drag coefficient of a knotted netting with 1-ply braided twines is larger than that of a knotless netting with 4-ply braided twines at an arbitrarily small attack angle. The drag coefficient of PE netting (solidity ratio = 0.187) with 2-ply braided twines is less than that of PA netting (solidity ratio = 0.185) with 4-ply braided twines for a similar solidity ratio and attack angle, indicating that the weave pattern and twine material, either one or both of them, significantly affect the drag coefficient. Under the same solidity ratio and attack angle, a PES netting with 4-ply braided twines has a relatively higher drag coefficient than a PES netting with 3-ply twisted twines, indicating that twisted netting has a higher resistance compared with braided netting.

### Model output results

The drag coefficient of a netting panel is strongly related to three categorical variables—knot type, twine material, and weave pattern—and three continuous variables—solidity ratio, Reynolds number, and attack angle (*P* < 0.05). The effects of small attack angle were significant in the drag coefficient model, and attack angle had a significant nonlinear effect on the drag coefficient (Table 1). The GAM results indicate that solidity ratio, Reynolds number, and attack angle have a nonlinear relationship with drag coefficient (EDF ≠ 1). A drag coefficient model was established to determine that the attack angle had the most impact on the drag coefficient of netting, followed by solidity ratio, weave pattern, Reynolds number, twine material, and knot type.

**Table 1:** Summary results of the GAMs for drag coefficients.

Variable	Estimate	Standard Error	t value	Pr(>|t|)	AIC
(Intercept)	1.70650	0.08742	19.521	<2e-16***	−7994.763
Knot type (knotted)	−0.61554	0.04975	−12.373	<2e-16***	−7994.763
Twine material (PE)	−0.37297	0.02467	−15.116	<2e-16***	−7988.802
Twine material (PES)	−3.85275	0.25632	−15.031	<2e-16***	
Weave pattern (2-ply braid)	−0.37297	0.02467	−15.116	<2e-16***	−6690.934
Weave pattern (2-ply twist)	3.69831	0.24847	14.884	<2e-16***	
Weave pattern (3-ply twist)	−0.37042	0.03793	−9.766	<2e-16***	
Weave pattern (4-ply braid)	−0.63288	0.04886	−12.952	<2e-16***	
**Variable**	**EDF**	**Ref.df**	**F**	**P-value**	**AIC**
Solidity ratio	5.998	6.000	817.8	<2e-16***	−6281.134
Attack angle	5.614	5.938	4017.7	<2e-16***	−3846.438
Reynolds number	4.982	5.628	130.3	<2e-16 ***	−7484.561

Note: The AIC value for this model as the designated factor was eliminated. With all the factors reserved, AIC is equal to −7994.763; deviance explained = 97.7%; R-sq. (adj.) = 0.977; *p*-values are presented for all significant (*p* < 0.05) single terms; estimated degree of freedom (EDF); reference degrees of freedom (Ref. df).

**Figure 6:**
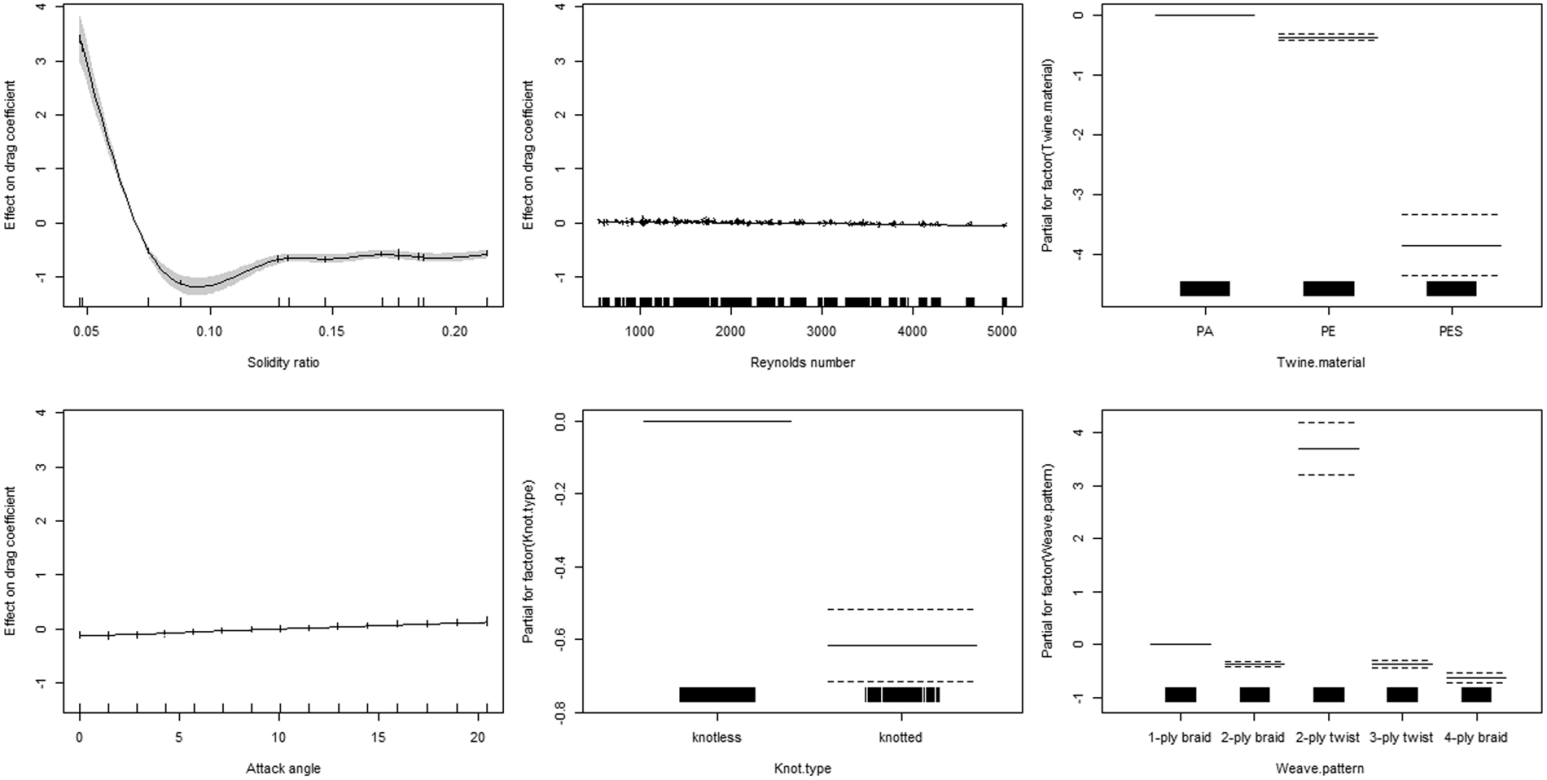
Estimated smoothness of 6 variables on the sinking depth of purse seine; y-axes show the partial effect of variable; shadow sections show the standard-error confidence intervals.

This drag coefficient model explained 97.7% of the total deviance. The GAM plots (rug plots) on the horizontal axis show the observed drag coefficient data points. The fitted function is shown by a thick line, and the dashed line and shade indicate 95% confidence bands (Fig. 6). When the solidity ratio was less than 0.1, the drag coefficient was almost inversely proportional to the solidity ratio. With the increase in solidity ratio from 0.1 to 0.213, the drag coefficient did not increase proportionally and remained constant, indicating that the excessively high solidity ratio did not contribute to the hydrodynamic coefficient of netting (Fig. 6). The drag coefficient decreased as the Reynolds number increased. The attack angle was positively correlated to drag coefficient, i.e., the drag coefficient of a netting panel at a lower attack angle was smaller than that of a netting at a higher attack angle (Fig. 6). A significant difference was observed in the drag coefficient between knotted and knotless nettings. Compared with PE and PA nettings, PES netting had a lower drag coefficient. The results also show that different weave patterns of twine have different effects on drag coefficient; 2-ply twisted twines had a greater effect.

### Empirical formula for PA drag coefficient

First, the drag coefficient formula for a knotless PA netting was derived based on Tauti’s theoretical formula, Kumazawa *et al*.’s^8^ formula, and Tang *et al*.’s^3^ formula to precisely estimate the hydrodynamic performance of a fishing gear at small attack angles. Considering the characteristic angle, the netting was set parallel to the flow, and a parallel drag coefficient was incorporated into the empirical formula. Using nonlinear regression with the data argument predicted from the formula of knotless netting panels with different solidity ratios, the following expression can be proposed:

2 $${C}_{D}=0.172{\alpha }^{-0.407}{{\rm{Re}}}^{-0.031}{(1+\sin {\theta }^{0.905})}^{1.822}$$

where, *C*_*D*_ is the drag coefficient of netting panel, α is the solidity ratio, *R*_*e*_ is Reynolds number, and *θ* is the attack angle.

**Figure 7:**
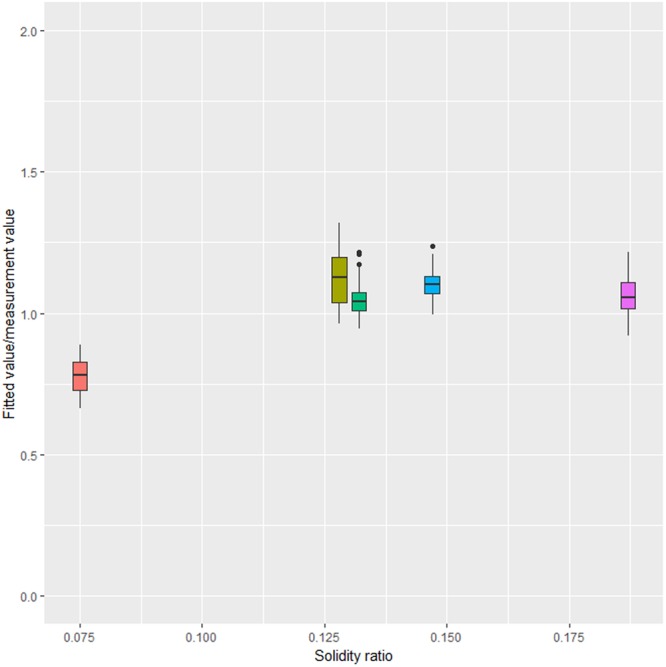
Comparison of fitted vs. measured values of drag coefficients for knotless PA nettings.

The residual standard error of nonlinear regression model (Eq. (2)) is 0.038. All parameters were tested to prove the reliability of nonlinear model by parameter validation testing (*P* < 0.05). The observed drag coefficient and fitted coefficient were compared (Fig. 7). In general, the ratio of fitted values to measured values is typically distributed at about 1, indicating that the nonlinear regression model of drag coefficient showed a suitable predication performance.

### Quantitative analysis

**Figure 8:**
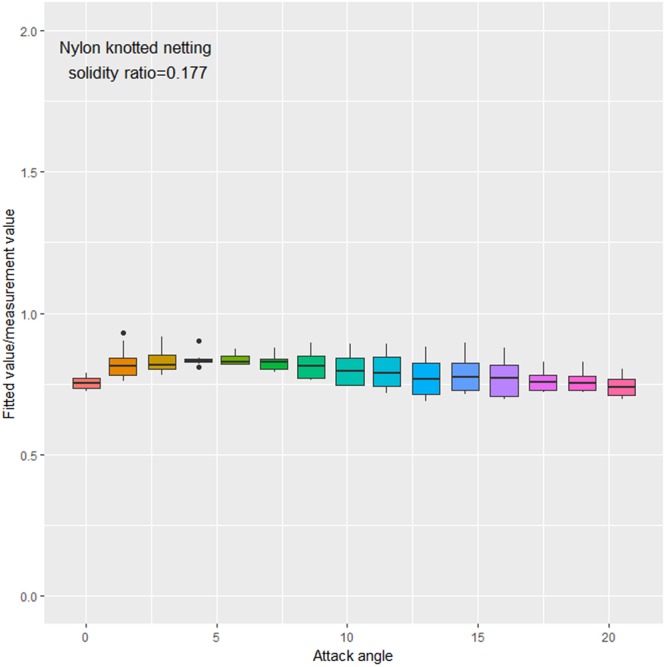
Comparison of fitted vs. measured values of drag coefficients for knotted PA nettings.

The drag coefficients of knotted PA netting were underestimated by ~21% at small attack angles, indicating that knot type is the most important factor affecting the hydrodynamic coefficient of a fishing net (Fig. 8). More remarkably, the number of twine plys of knotted netting is less than that of knotless netting, which would also deviate the results.

**Figure 9:**
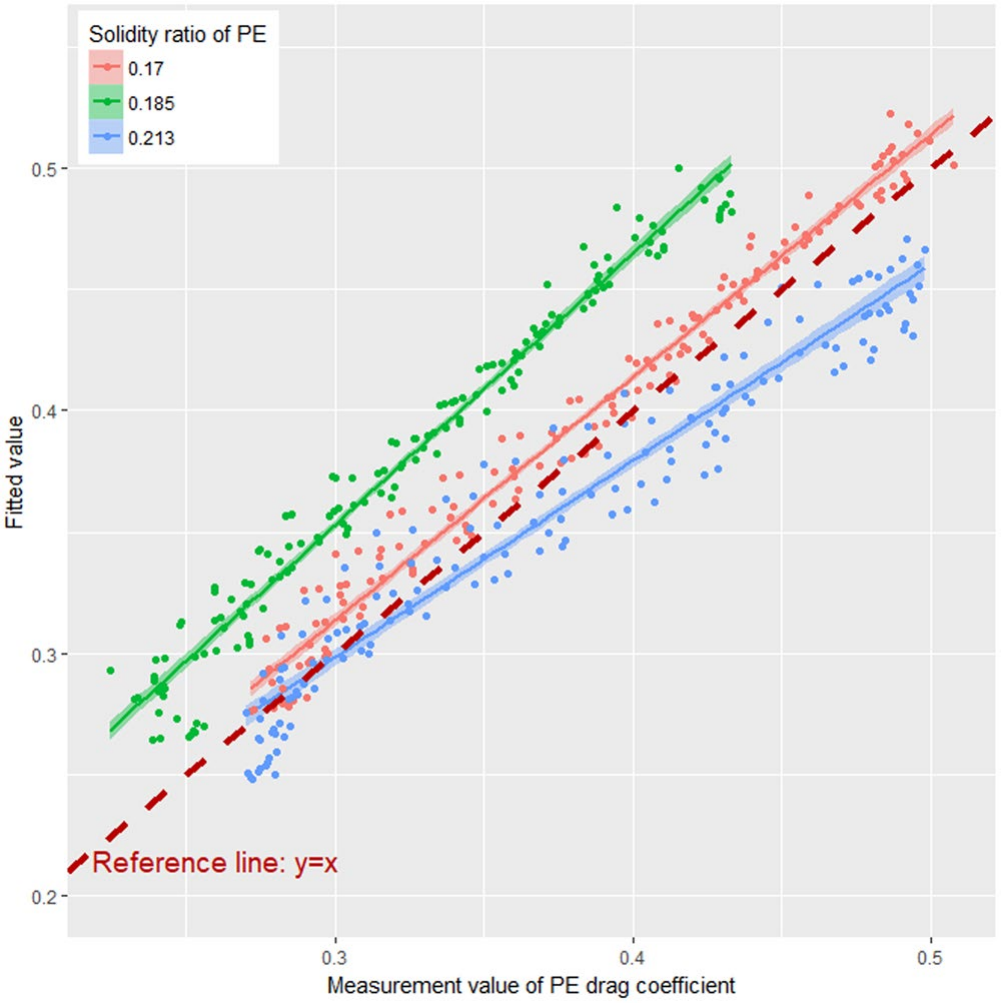
Comparison of fitted vs. measured values of drag coefficients for PE nettings.

A fitted drag coefficient of PE can be calculated using formula (2) and compared with the measured value obtained from flume experiment. Notably, here the fitted value refers to the drag coefficient of knotless PA netting with the same solidity ratio as PE netting. This increased the quantitative difference in drag coefficient between knotless PE and PA nettings. The measurement-fitted slope of PE netting was smaller than that of reference line, and the drag coefficient of 2-ply braided knotless PE netting was ~8.4% higher than that of 4-ply braided knotless PA netting at average level (Fig. 9). This can be attributed to the differences in weave pattern and twine material of the two types of nettings.

**Figure 10:**
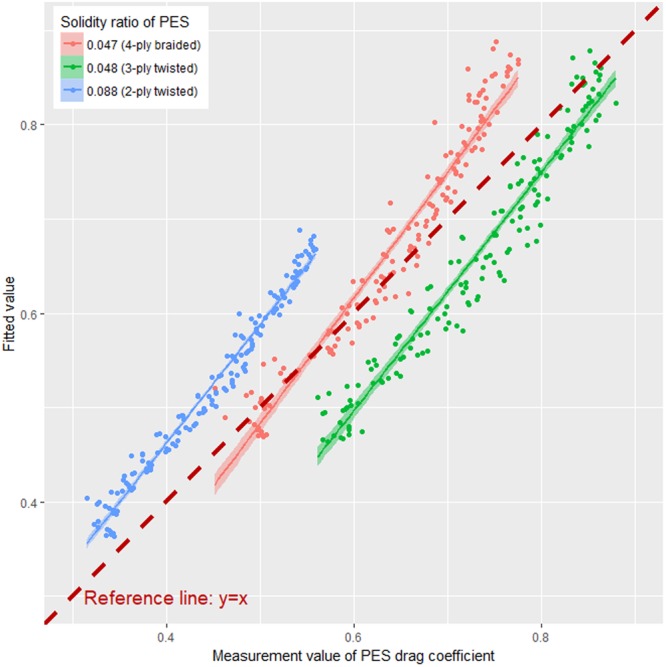
Comparison of fitted vs. measured values of drag coefficients for PES nettings.

According to the calculations, the drag coefficient of knotless PES netting was ~7% higher than that of 4-ply braided knotless PA netting (Fig. 10). Most notably, each PES netting had different weave patterns. Moreover, the drag coefficient of 4-ply braided PES netting was 14.6% higher than that of 3-ply twisted PES netting. This result once again shows that the braided netting had a higher hydrodynamic force.

## Discussion

The problems of hydrodynamic characteristics of nettings at small attack angles have historically been a difficult problem to solve because of experimental frame and setup limitations. A self-designed setup was used to measure the hydrodynamic forces of netting panels made of three materials at small attack angles. The experimental frame with a low resistance is the basic demand of netting hydrodynamic experiments. The resistance caused by a frame without a netting panel occupied 13.6% of the total resistance. The frame stability was also evaluated under different attack angle conditions. The ratio of frame resistance to total resistance was less than 20%, and the ratio decreased with the increase in attack angle. In term of resistance, the frame satisfied the experiment requirements.

The hydrodynamic coefficients of nettings are affected by many factors such as solidity ratio, Reynolds number, and attack angle. The nonlinear relationships are affected by the drag coefficient at small attack angles. In this respect, our results are similar to those reported by Kumazawa *et al*.^8^, Zhou *et al*.^10^, and Tang *et al*.^3^. The drag coefficients of nettings made of three materials decreased with the increase in solidity ratio at small attack angles; this can be attributed to the “shielding effect” as the fluid is transferred from upstream to downstream strands. The solidity ratio is considered as the main factor affecting the shielding caused by the netting panel. Bi *et al*.^12^ found a significant reduction in flow velocity downstream from the netting panel, and the shielding effect increased with increasing netting solidity ratio, i.e., with increasing solidity ratio, the flow velocity on downstream netting decreased significantly, leading to a relatively smaller force on the netting panel^3,12^. In addition, a nominally high solidity ratio was used to calculate the drag coefficient in the case of small attack angles, also resulting in a low value.

The decrease in flow velocity increased with increasing inclination angle between the netting panel and vertical direction^12,13^. It is important to emphasize that inclination angle is not the attack angle mentioned in this paper. In fact, there is a positive and reciprocal relationship between inclination angle and attack angle (an inclination angle of 0° corresponds to an attack angle of 90°). The netting solidity increased along the flow direction as the attack angle decreased, and the shielding effect increased. Therefore, the shielding effect is considered the maximum when the netting panel is set parallel to flow^10^. This explains why the drag coefficient is nonlinearly and positively related to attack angle.

For a netting panel, the flow velocity determines the Reynolds number by affecting the flow pattern around the netting. The drag coefficient reached a steady value for flow velocities of 50–60 cm/s and remained approximately constant until the highest experimental velocity was reached. This is due to the transition of flow regime from a viscous laminar flow at low velocities (Re < 2000) to developed turbulent flow at higher velocities^2^. This also explains the sharp change in drag coefficient when the Reynolds number ranged from 0 to 2000 as shown in Fig. 4.

It was confirmed that knot type, twine material, and weave pattern clearly affect the drag coefficient (*P* < 0.05). The quantitative analysis results show that the average drag coefficient of knotless PA netting panel was ~79% of that of knotted netting. The effect of knot on drag coefficient increased as the attack angle increased (Fig. 8). This can be attributed to the added project area in flow direction, increasing the force acting on netting, especially knotted netting. The meshes used in fishing gear have knots asymmetric to the netting plane, a characteristic that produces a low hydrodynamic force when the netting has a zero attack angle along the flow direction^14^. The knots show either a positive or negative attack angle in horizontal panels, producing drag forces and changes in system geometry in most cases for trawls. Broadhurst *et al*.^14^ quantified the drag forces in a flume tank using four prawn trawls with all possible combinations of knot orientation in the bottom and top panels; two trawls with their top panels producing a negative attack angle had up to 10% less total drag compared with the two trawls with their top panels with a positive attack angle. Knot orientation is a simple technical factor that affects the engineering performance of a trawl^15^.

In terms of hydrodynamic characteristics, the difference in twine materials is mainly manifested in water imbibition, hydroscopic property, and netting-surface roughness. Among them, water imbibition is one of the most important material performance that affects the hydrodynamic coefficient of a fishing net. PA netting with better water imbibition property makes it easy to flow water through the netting, resulting in a low water resistance compared with PE netting with weak water imbibition. This study also confirmed that the drag coefficient of PE netting was higher than 8.4% of PA netting in the case of small attack angles.

Two types of weave patterns, braided and twisted, are commonly used in modern fishing gear. Different weave patterns change the way of water flow, resulting in different flow resistances. For the same solidity ratio of PES netting, a 14.6% difference was observed in the drag coefficients between braided and twisted nettings. The interstices between strands for braided netting were smaller than twined netting, and the water did not flow easily through the netting, causing a large water resistance.

The small attack angle formula proposed in this paper was compared with the literature, the estimated drag coefficients by Aarsnes *et al*.^9^ and Hosseini *et al*.^7^ were lower than those observed in this study. The inclined drag coefficient reported by Kumazawa *et al*.^8^ was slightly higher than ours. It was due to the differences in netting samples, experimental setup, and hypothetical mathematical model. Tang *et al*.^3^ applied a cylindrical frame to test the drag force of netting. Their results are consistent with the results of this study at attack angles ranging from 0° to 10°, but a difference was observed at attack angles ranging from 10° to 20°. The use of a cylindrical frame caused turbulence at a higher flow velocity and provided inaccurate measurement results^3^. This is an important reason for the abovementioned difference.

For small attack angles, the flow characteristics through and around a netting panel can be quantified using particle image velocimetry (PIV), acoustic Doppler velocimeter (ADV), and computational fluid dynamics (CFD)^12,13,16^. This helps us to calculate the hydrodynamic force and understand the hydrodynamic characteristics in the design and modification of net. Because simple modifications such as knots, materials, and inclination angles within the existing configurations of fishing gear are likely to be more easily accepted than completely alternate designs, they should be assessed as a starting point for improving the engineering efficiency.

## Materials and Methods

All the flume tank experiments were carried out in a flume tank at the Tokyo University of Marine Science and Technology (TUMST), Tokyo, Japan. The flume tank was 9.0 m in length, 2.2 m in width, and 1.6 m in depth. A pump located at one end of the flume tank was used to generate a steady flow and regulate the low velocity by changing its rotation frequency.

### Experimental netting

The hydrodynamic coefficient was determined for three knotless PE nettings, three knotless PES nettings, five knotless PA nettings, and one knotted PA netting. All the netting panels were fabricated by Nichimo Co. (Japan). The characteristics of netting panel samples are shown in Table 2. The net solidity ratio is the ratio between the projected area of a netting panel and outline area, referring to the total area enclosed by the netting panel^2,4^. For a netting panel, the net solidity can be expressed as follows:

3 $$\alpha =\frac{d(2l\pm d)}{{l}^{2}\,\sin \,2\phi }$$

where, *d* is the twine diameter, *l* is the bar length, *φ* is the mesh opening angle, “+” represents knotted netting, and “_” represents knotless netting. The mesh opening angle was maintained at 45° when the netting was attached to a frame in the experiments.

**Table 2:** Structural parameters of netting panels used in this study arranged according to their solidity ratio.

Netting No.	Twine materials	Knot type	Bar diameter (mm)	Bar length(mm)	Solidity ratio	Weave pattern
net-1	PA	knotless	1.96	52.87	0.075	4-ply braided
net-2	PA	knotless	3.22	48.47	0.128	4-ply braided
net-3	PA	knotless	3.17	46.87	0.132	4-ply braided
net-4	PA	knotless	3.65	48.40	0.147	4-ply braided
net-5	PA	knotless	4.47	45.40	0.187	4-ply braided
net-6	PA	knotted	3.67	43.13	0.177	1-ply braided
net-7	PE	knotless	3.67	38.30	0.185	2-ply braided
net-8	PE	knotless	2.70	30.80	0.170	2-ply braided
net-9	PE	knotless	4.13	36.60	0.213	2-ply braided
net-10	PES	knotless	2.13	90.53	0.047	4-ply braided
net-11	PES	knotless	2.25	93.33	0.048	3-ply twisted
net-12	PES	knotless	2.12	47.20	0.088	2-ply twisted

### Hydrodynamic coefficient

The dimensionless coefficients *C*_*D*_ (drag coefficient) and *C*_*L*_ (lift coefficient) of a plane netting were calculated using the following equations^8,17^:

4 $${C}_{D}=\frac{2{F}_{D}}{\rho \alpha {S}_{0}{V}^{2}}$$

5 $${C}_{L}=\frac{2{F}_{L}}{\rho \alpha {S}_{0}{V}^{2}}$$

where *F*_*D*_ and *F*_*L*_ are the measured drag and lift forces acting on the netting, *ρ* is density of water, *V* is the flow velocity, *α* is the solidity ratio, *S*_0_ is the outline area of netting panel, and *αS*_0_ is the projected area of netting panel.

### Experimental setup

**Figure 11:**
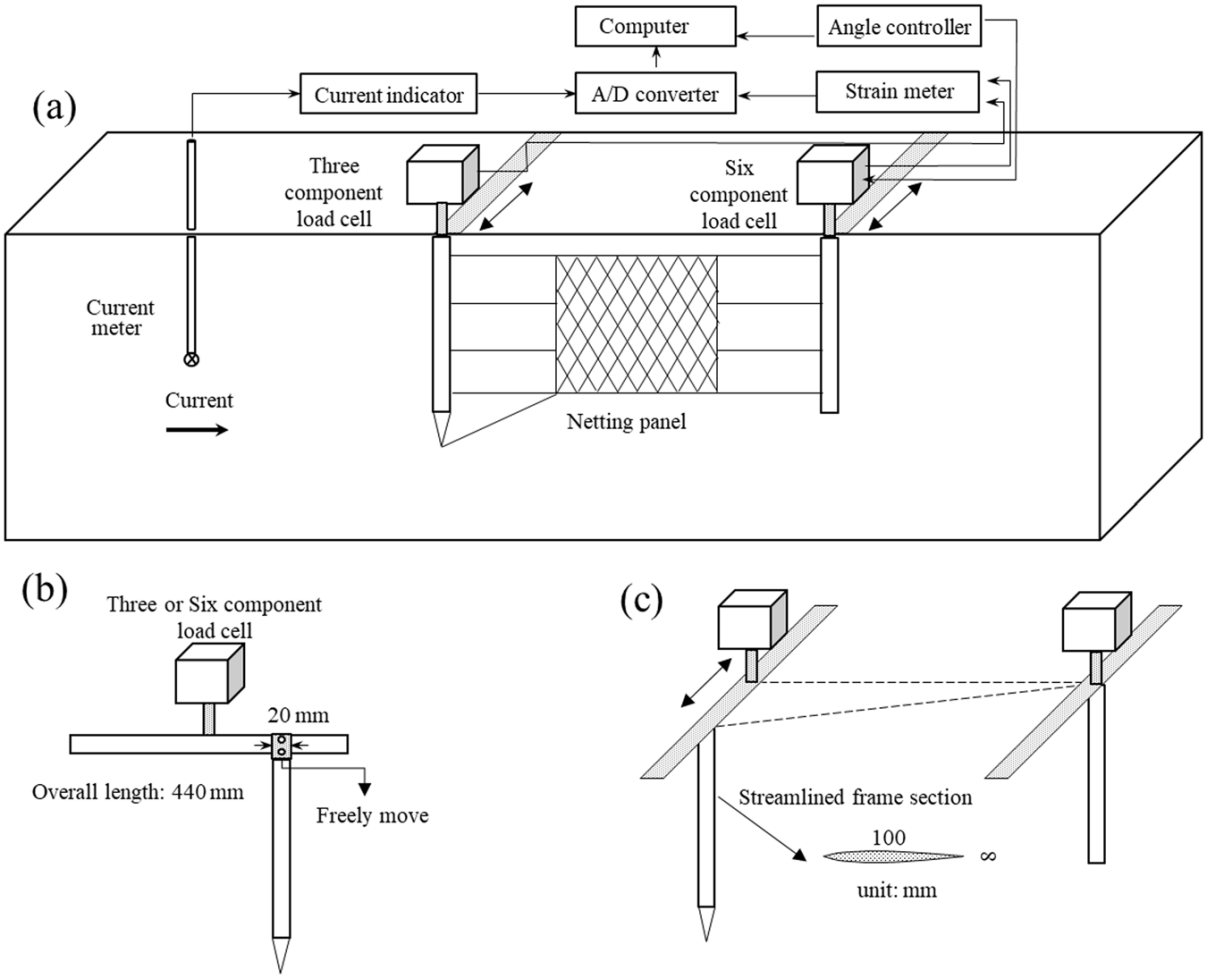
Schematic diagram of experimental apparatus for hydrodynamic experiments of netting panels at small attack angles: (**a**) overall experimental frame, (**b**) single streamlined plane assembly, and (**c**) attack angle adjustment.

Effective experimental frames and setups are essential for measuring the hydrodynamic force acting on a netting panel. Therefore, the test device has two requirements: The drag needed for this frame is lower than that needed for experimental netting; the frame can have a steady drag but not produce lift at different attack angles. Tang *et al*.^3^ reported that application of a streamlined frame prevented turbulence or vortices to experimental netting by flow velocity and exhibited a lower frame drag. Finally, two streamlined planes were used to design the main structure of frame, and some thin steel wires were used to fix the basic frame (Fig. 11). According to the condition of TUMST flume tank, a specialized experimental setup was designed to measure the drag and lift of netting panels at small attack angles, as shown in Fig. 11.

A streamlined plane (100 mm in length, 8 mm in thickness, and weight of 1.075 kg) was attached to the force measuring instrument using a steel plane with a length of 440 mm, width of 40 mm, and weight of 1.225 kg. A groove (slider) with a width of 20 mm was fixed on the top of streamlined plane and used to fasten the steel plane (Fig. 11b). The lateral distance can be changed by moving the slider that varies the attack angle (Fig. 11c). A total of 22 inclination angles were set up for experiments (Table 3). Some thin wires were used to assemble experimental netting panels on the frame and compose the streamlined frame structure (Fig. 11a). After the frame was assembled and placed in three- and six-component load cells (5 kgf, Denshi Kogyo Co., Japan) with screws, still a large amount of configuration adjustment was required. The special measuring frame design placed two streamlined planes in stretch tight and parallel state along the incoming flow. The flow velocity was measured using a propeller tachometer located at 1.5 m in front of the frame; the flow velocity ranged from 30 cm/s to 120 cm/s and increased at 10 cm/s interval. The data for each experiment were gathered at 20 Hz over a period of 20 s using a data acquisition system.

**Table 3:** Inclination angles of experimental design.

Horizontal movement (cm)	Sine value	Attack angle	Vertical distance (cm)
2	0.025	1.433	79.975
4	0.050	2.866	79.900
6	0.075	4.301	79.775
8	0.100	5.739	79.599
10	0.125	7.181	79.373
12	0.150	8.627	79.095
14	0.175	10.079	78.765
16	0.200	11.537	78.384
18	0.225	13.003	77.949
20	0.250	14.478	77.460
22	0.275	15.962	76.916
24	0.300	17.458	76.315
26	0.325	18.966	75.657
28	0.350	20.487	74.940
30	0.375	22.024	74.162
32	0.400	23.578	73.321
34	0.425	25.151	72.415
36	0.450	26.744	71.442
38	0.475	28.359	70.399
40	0.500	30.000	69.282
42	0.525	31.668	68.088

The measuring methods and steps of lift and drag are as follows:

1. The voltage signals of drag and lift forces are transmitted to the strain meter using the sensor when the flow passes thought the experimental frame and netting (Fig. 11a).
2. The strain meter amplifies and integrates the signals from the sensors, producing analog signal information that can be displayed continuously online11.
3. The A/D converter changed analog signals to digital signals.
4. The drag and lift forces are shown using calibration coefficients on computer. In the above manner, the flow speed signals also are obtained from the propeller tachometer sensor.

### Data analysis

Generalized additive models (GAMs) using an identity link function with Gaussian error distribution were used to evaluate the effects of various factors (twine materials, knot types, Reynolds number, solidity ratio, attack angle, and weave pattern) on the drag coefficients of netting panels made of different materials. The GAMs are regression models; the linear predictor depends on unknown smoothing functions of some predictor variables instead of linear coefficients as covariates^18^. The GAMs can be expressed as follows:

6 $${C}_{D} \sim s({R}_{e})+s({S}_{r})+s(\theta )+K+M+W+\varepsilon $$

where, *C*_*D*_ is the drag coefficient of netting panel, *R*_*e*_ is Reynolds number, *S*_*r*_ is the solidity ratio, *θ* is the attack angle, *K* is the knot type, *M* is the twine material, and *W* is the weave pattern. Functions *s* () are one-dimensional smooth functions of variables. Functions *s* () are the categorical functions of variables *K*, *M*, and *W* are the categorical variables. *ε* is the residual error subject to normal distribution (E (*ε*) = 0, *ε* = *σ*^2^).

To prevent model overfitting, the maximum degree of freedom was set at 7(k = 7) for univariate terms. Chi-square statistical significance tests and Akaike information criteria (AIC) were used as the model selection criteria. A stepwise backward selection was applied to identify the optimal model. All the statistical analyses were conducted in the R programming environment. GAMs were built and fitted using the mgcv package.

## Electronic supplementary material

Experiment data

## Data Availability

The datasets generated during and/or analyzed during the current study are available from the corresponding author on reasonable request.

## References

[1] Sterling D, Balash C (2017). Engineering and catching performance of five netting materials in commercial prawn-trawl systems. Fisheries Research.

[2] Tsukrov I, Drach A, DeCew J, Swift MR, Celikkol B (2011). Characterization of geometry and normal drag coefficients of copper nets. Ocean Engineering.

[3] Tang H (2017). The effect of netting solidity ratio and inclined angle on the hydrodynamic characteristics of knotless polyethylene netting. Journal of Ocean University of China.

[4] Tsukrov I, Eroshkin O, Fredriksson D, Swift MR, Celikkol B (2003). Finite element modeling of net panels using a consistent net element. Ocean Engineering.

[5] Zhan JM (2006). Analytical and experimental investigation of drag on nets of fish cages. Aquacultural Engineering.

[6] Balash C, Colbourne B, Bose N, Raman-Nair W (2009). Aquaculture net drag force and added mass. Aquacultural Engineering.

[7] Hosseini SA, Lee CW, Kim HS, Lee JH, Lee GH (2011). The sinking performance of the tuna purse seine gear with large-meshed panels using numerical method. Fisheries Science.

[8] Kumazawa T, Hu FX, Kinoshita H, Tokai T (2012). Hydrodynamic characteristics of plane minnow netting made of high-strength polyethylene (Dyneema). Nippon Suisan Gakkaishi.

[9] Aarsnes, J.V., Rudi, H. & Løland, G. Current forces on cage, net deflection. In *Engineering for Offshore Fish Farming*. (Thomas Telford, London, 137–152, 1990).

[10] Zhou C, Xu LX, Hu FX, Qu XY (2015). Hydrodynamic characteristics of knotless nylon netting normal to free stream and effect of inclination. Ocean Engineering.

[11] Tang H, Xu L, Hu F (2018). Hydrodynamic characteristics of knotted and knotless purse seine netting panels as determined in a flume tank. PLoS ONE.

[12] Bi CW, Zhao YP, Dong GH, Xu TJ, Gui FK (2013). Experimental investigation of the reduction in flow velocity downstream from a fishing net. Aquacultural engineering.

[13] Zhao YP (2013). Numerical simulation of the flow around fishing plane nets using the porous media model. Ocean Engineering.

[14] Broadhurst MK, Balash C, Sterling DJ, Millar RB, Matsubara S (2017). Effects of knot orientation on the height and drag of a penaeid trawl. Fisheries Research.

[15] Broadhurst MK, Suuronen P, Hulme A (2006). Estimating collateral mortality from towed fishing gear. Fish and Fisheries.

[16] Patursson Ø (2010). Development of a porous media model with application to flow through and around a net panel. Ocean Engineering.

[17] Kawakami, T. The theory of designing and testing fishing nets in model. In *Modern Fishing Gear of the World*, **2** (Fishing News (Books), London, 471–482, 1964).

[18] Harley SJ, Suter JM (2007). The potential use of time-area closures to reduce catches of bigeye tuna (*Thunnus obesus*) in the purse-seine fishery of the eastern Pacific Ocean. Fishery Bulletin.

